# Intervention with isoleucine or valine corrects hyperinsulinemia and reduces intrahepatic diacylglycerols, liver steatosis, and inflammation in Ldlr−/−.Leiden mice with manifest obesity‐associated NASH

**DOI:** 10.1096/fj.202200111R

**Published:** 2022-07-13

**Authors:** Eveline Gart, Wim van Duyvenvoorde, Martien P. M. Caspers, Nikki van Trigt, Jessica Snabel, Aswin Menke, Jaap Keijer, Kanita Salic, Martine C. Morrison, Robert Kleemann

**Affiliations:** ^1^ Department of Metabolic Health Research The Netherlands Organization for Applied Scientific Research (TNO) Leiden the Netherlands; ^2^ Human and Animal Physiology Wageningen University Wageningen The Netherlands; ^3^ Department of Microbiology and Systems Biology The Netherlands Organization for Applied Scientific Research (TNO) Zeist the Netherlands; ^4^ Department of Vascular Surgery Leiden University Medical Center Leiden the Netherlands

**Keywords:** branched‐chain amino acids, diacylglycerols, insulin resistance, lipid metabolism, non‐alcoholic steatohepatitis, oxidative stress

## Abstract

Non‐alcoholic steatohepatitis (NASH) is associated with a disturbed metabolism in liver, insulin resistance, and excessive accumulation of ectopic fat. Branched‐chain amino acids (BCAAs) may beneficially modulate hepatic lipids, however, it remains unclear whether individual BCAAs can attenuate already established NASH and associated oxidative‐inflammatory stress. After a 26 weeks run‐in on fast food diet (FFD), obese Ldlr−/−.Leiden mice were treated for another 12 weeks with either valine or isoleucine (3% of FFD) and then compared to FFD controls. Valine and isoleucine did not affect obesity, dyslipidemia, gut permeability, or fecal fatty acid excretion, but significantly reduced hyperinsulinemia. Valine and isoleucine reduced ALT, CK18‐M30, and liver steatosis with a particularly pronounced suppression of the microvesicular component (−61% by valine and −71% by isoleucine). Both BCAAs decreased intrahepatic diacylglycerols and 4‐hydroxynonenal immunoreactivity, a marker for oxidative stress‐induced lipid peroxidation. Functional genomics analysis demonstrated that valine and isoleucine affected BCAA metabolism genes, deactivated master regulators of anabolic pathways related to steatosis (e.g., SREBPF1), and activated master regulators of mitochondrial biogenesis (e.g., PPARGC1A) and lipid catabolism (e.g., ACOX1, AMPK). This correction of critical metabolic pathways on gene expression level was accompanied by a significant decrease in histological liver inflammation, and suppression of FFD‐stimulated cytokine and chemokine proteins KC/CXCL1, MCP‐1/CCL2, and MIP‐2/CXCL2 and their pathways. In conclusion, dietary intervention with either valine or isoleucine corrected liver diacylglycerols, gene expression of multiple metabolic processes, and reduced NASH histology with profound hepatoprotective effects on oxidative stress and inflammatory proteins.

## 1 INTRODUCTION

Non‐alcoholic fatty liver disease (NAFLD) is the hepatic manifestation of the metabolic syndrome, which is characterized by obesity, dyslipidemia, hyperinsulinemia, and insulin resistance.^1^, ^2^ The strong association between NAFLD, insulin resistance, and dyslipidemia is thought to be causal to the increased risk of atherosclerosis in NAFLD patients.^3^ The relationship between hyperinsulinemia NAFLD and atherosclerosis has been explained by an insulin‐mediated upregulation of SREBF1 (*SREBP‐1c*). This upregulation stimulates hepatic de novo fatty acid (FA) synthesis^4^, ^5^ and thereby the excessive accumulation of fat (steatosis), while at the same time promoting hepatic VLDL secretion and dyslipidemia. Hepatic steatosis in NAFLD is typically associated with disturbed lipid handling processes among which are increased FA synthesis and diminished FA oxidation.^6^ These disturbances in lipid handling are also associated with hepatic insulin resistance mediated by diacylglycerols (DAGs),^7^ increased oxidative stress, and hepatocellular damage.^8^, ^9^ Together they promote the development of non‐alcoholic steatohepatitis (NASH) characterized by liver inflammation and activated inflammatory cytokine signaling pathways stimulated by TNF‐α, IL‐1β, KC/CXCL1, MCP1/CCL2, and others.^10^ In particular the accumulation of small lipid droplets in hepatocytes, referred to as microvesicular steatosis, is thought to be related to impaired lipid processing and mitochondrial dysfunction.^11^ Also, deactivation of AMP‐activated protein kinase (AMPK), a major regulator of energy homeostasis and lipid catabolism, is frequently observed in NASH.^12^, ^13^ This deactivation drives anabolic processes including lipogenesis via SREBF1 while impairing peroxisomal β‐oxidation (e.g., ACOX1 activity).

Therapeutic strategies for NAFLD/NASH that have been developed during the past several years do not or only partially correct the aforementioned complex disturbances of liver lipid metabolism.^9^ Hence, there is a great interest in new approaches, including nutritional treatments, that may restore metabolic homeostasis and reduce liver inflammation. A recent study showed that a mix of equal amounts of the branched‐chain amino acids (BCAAs) leucine, isoleucine, and valine administered via the diet, can prevent hepatic steatosis in mice fed a choline‐deficient high‐fat diet.^14^ However, choline deficiency is a physiologically artificial condition and is mainly used experimentally to inhibit the assembly and secretion of VLDL particles in the liver.^15^ As a consequence, also the development of dyslipidemia is prevented under such experimental conditions which contrasts with the situation in NASH patients. Furthermore, it is unclear how these BCAA effects are achieved and studies investigating whether individual BCAA can correct the multiple metabolic pathways affected in NASH have not been performed.

It is also unknown whether the individual BCAAs can attenuate NASH development in a therapeutic setting, i.e., starting treatment when obesity, dyslipidemia, hyperinsulinemia, and liver steatosis are already established. To study the potential hepatoprotective effects of therapeutic intervention with single BCAAs, we employed dietary supplementation with valine or isoleucine in an established mouse model for diet‐induced NAFLD/NASH with atherosclerosis, Ldlr−/−.Leiden mice.^16^, ^17^, ^18^, ^19^ These mice exhibit hyperinsulinemia, obesity, and human‐like pathological hallmarks of NASH^20^ in combination with hepatocellular damage from oxidative stress.^16^ These mice also express key inflammatory pathways observed in NASH patients^18^, ^19^ when fed a fast‐food diet (FFD) with a macronutrient composition and cholesterol content comparable to human diets.^21^ Next‐generation sequencing (NGS) and ingenuity pathway analysis provide a possibility to comprehensively analyze metabolic and inflammatory pathways including their upstream regulators based on integrated gene expression analysis of downstream target genes. We employed this technique in conjunction with hepatic protein analysis by Western blot and multiplex assays to support histological and immunohistochemical observations made in the liver and to identify the metabolic processes and inflammatory pathways that were affected by the individual BCAAs.

## 2 MATERIALS AND METHODS

### 2.1 Animal experiment

The animal experiment was performed according to standards of the Animal Care and Use Committee and ethically approved by an independent Animal Welfare Body (IVD TNO; approval number 20172064/TNO‐439) and the ARRIVE guidelines were followed. Male Ldlr−/−.Leiden mice (genetic background 94% C57BL/6J and 6% 129S) obtained from the breeding stock at TNO Metabolic Health Research (Leiden, the Netherlands) were group‐housed (four to five mice per cage) in Macrolon cages in clean‐conventional animal rooms (relative humidity 50%–60%, temperature ~21°C, light cycle 7 a.m. to 7 p.m.) in an animal facility accredited by the American Association for Accreditation of Laboratory Animal Care (AAALAC) at TNO Metabolic Health Research with ad libitum access to food and water.

During a 26‐week run‐in period, three groups of mice were fed a fast‐food diet (FFD; 41 kcal% fat from milk fat, 44 kcal% from primarily fructose as carbohydrate, and 14 kcal% from mainly casein as a protein source; Research Diets, New Brunswick, NJ, USA). After 26 weeks, two FFD groups started with a 12‐week intervention in which FFD was supplemented with 3% (w/w) valine (FFD + Val) or isoleucine (FFD + Iso), and these two treatment groups were compared to a FFD control group. Mice were matched into these three treatment groups of *n* = 15 mice per group, based on body weight, plasma cholesterol, and triglycerides and blood glucose. *N* = 15 mice are required to detect statistical differences in NASH (at *α* = 0.05 and power = 0.8), the main end‐point. The concentration of valine and isoleucine was based on a long‐term dietary supplementation study with BCAAs.^22^ Of note, in the BCAA intervention groups the casein content was lowered to make sure that the intake of total protein was comparable between the groups. In addition, levels of other essential amino acids were supplemented to bring them back up to the same level as in the FFD control group. Adjustments were made following the guidelines of the National research council, and the requirements put forward by the subcommittee on laboratory animal nutrition.^23^ During the whole study period of 26 weeks run‐in plus 12 weeks of intervention, thus 38 weeks, a reference group (*n* = 8) remained on a low‐fat chow diet (chow; Sniff‐R/M‐V1530, Uden, the Netherlands).

Blood samples were obtained from the tail vein in 5‐h‐fasted mice, and body weight, food intake, and body composition measurements were acquired at set time points during the study. After 38 weeks mice were sacrificed by gradual‐fill CO_2_ exposure and liver tissues were collected for biochemical measurements and histopathology (snap‐frozen and formalin fixation, respectively). Effects of the valine and isoleucine interventions were evaluated using histological and biochemical techniques on features of the metabolic syndrome and NASH. To gain insight into the molecular processes affected by valine and isoleucine, we performed NGS and subsequent bioinformatical analysis to study entire metabolic and inflammatory pathways and the respective activity of upstream regulators.

### 2.2 Blood chemistry

Analysis of cholesterol, triglycerides (TGs), insulin, alanine aminotransferase (ALT), tissue inhibitor of metalloproteinases‐1 (TIMP‐1) in EDTA plasma, and glucose in whole blood was performed as described previously.^24^ In addition, cytokeratin 18‐M30 (CK18‐M30) and apolipoprotein B (ApoB) were measured in EDTA plasma according to the manufacturer's instructions (Cusabio CSB‐E14265m, Houston, USA, and Abcam Ab230932, Cambridge, UK, respectively).

For lipoprotein profiles analysis, lipoproteins were first separated via fast protein liquid chromatography (FPLC) using an AKTA apparatus (Pharmacia, Roosendaal, the Netherlands), as previously described.^25^, ^26^ Subsequently, total cholesterol and triglycerides were measured in the fractions collected for profiling with enzymatic assays (Roche diagnostics, Basel, CHF).

Plasma amino acids were extracted and derivatized with AccQTag reagent (Waters). The derivatized amino acids were then analyzed by LC–MS. For quantification of the 20 normal amino acids (including the branched‐chain amino acids), calibration curve standards prepared in albumin solution were measured. Nitrogen‐15 and carbon‐13 amino acid internal standards were added to all samples before extraction.

### 2.3 Fecal fatty acid excretion

Fatty acid excretion was determined in feces collected from cages over a 3‐day time period. These feces were freeze‐dried and weighed. An aliquot of these lyophilized feces (15 mg) was derivatized as previously reported^27^ using pentadecanoic acid (C15:0) as the internal standard. The samples were prepared as previously described^27^ and applied on a GC column (CP Sil88, Chrompack International) in a Scion 436‐GC (Goes, the Netherlands) equipped with a flame ionization detector. Quantification of the fecal fatty acids (C14:0, C16:0, C16:1, C18:0, C18:1, C18:2, C18:3, as these are the major FA present) was based on the area ratio to the internal standard and expressed as μmol/mouse/day.

### 2.4 Liver histopathology

NASH was scored in hematoxylin–eosin‐stained cross‐sections based on the human NAS scoring system using a standardized method for rodents.^20^ Briefly, for each mouse, the percentage of the total liver section affected by steatosis (macrovesicular and microvesicular) and hypertrophy (abnormally enlarged hepatocytes) was determined. Hepatic inflammation was quantified by counting the number of inflammatory aggregates in 5 fields per mouse at 100× magnification (field of view 4.15 mm^2^) and expressed as the number of aggregates per mm^2^ as reported.^24^

Oxidative stress‐induced lipid peroxidation marker 4‐hydroxynonenal (4‐HNE) was analyzed in 4‐HNE‐stained liver sections (with Rabbit anti‐4‐HNE 1:2000, ref.393207, Millipore Corporation, Billerica, MA, USA PBS) as previously described,^28^ for *n* = 5 chow, and *n* = 8 FFD, FFD + valine and FFD + isoleucine. 4‐HNE‐positive immunoreactive structures were counted in three non‐overlapping fields and expressed per mm^2^.

### 2.5 Intrahepatic diacylglycerol content determination

Diacylglycerol (DAG) content was quantified in crude liver homogenates according to the manufacturer's instructions (ab242293, Abcam, Cambridge, UK). In short, this assay extracts cellular lipids using methanol, chloroform, and NaCl. Extracted lipids are treated with a kinase that phosphorylates DAGs to yield phosphatidic acid (PA). A lipase then hydrolyzes PA into glycerol‐3‐phosphate, which is subsequently oxidized by glycerol‐3‐phosphate oxidase (GPO) to produce hydrogen peroxide that reacts with a fluorometric probe.

### 2.6 Hepatic genome‐wide gene expression analysis

Briefly, liver total RNA was isolated using RNA‐Bee (Bio‐Connect, Huissen, the Netherlands) and purified using PureLink RNA Mini Kit (Thermo Fisher Scientific, Waltman, USA). RNA concentration was determined spectrophotometrically using the Nanodrop 1000 (Isogen Life Science, De Meern, the Netherlands), and RNA quality was assessed using the 2100 Bioanalyzer (Agilent Technologies, Amstelveen, the Netherlands). Strand‐specific messenger RNA sequencing libraries for the Illumina (llumina NovaSeq6000, San Diego, CA) platform were generated paired‐end 150 bp for approximately 20 million paired‐End reads per sample at Genomescan (Leiden, the Netherlands).

The sequences were filtered, trimmed, and subjected to quality control as described previously.^29^ These files were then merged and aligned to the reference genome “Mus_musculus.GRCm38.gencode.vM19”. To count the read mapping frequency per gene Htseq‐count 0.6.1p1 was used, and the resulting count files served as input for the differentially expressed genes (DEGs) analysis using the Deseq2‐method.^30^ DEGs were used as an input for pathway and upstream regulator analysis through Ingenuity Pathway Analysis (IPA).^31^ IPA also uses gene expression data of all known downstream target genes to predict the activation or deactivation of an upstream regulator as reported.^24^

### 2.7 Hepatic protein expression analysis

Protein expression of phosphorylated‐BCKDH, total‐BCKDH, phosphorylated‐AMPK, and tubulin was determined using Western blot analysis. Liver homogenates were made with ice‐cold lysis buffer as previously described,^24^ and protein content was determined (BCA Protein Assay Kit, Thermo Fisher Scientific). Protein samples (40 μg) were separated and blotted as previously reported.^24^ The blotting membranes were treated with block buffer for 1 h (5% (*w*/*v*) milk powder in tris‐buffered saline with 0.1% Tween 20 (TBST)) and incubated overnight at 4°C in block buffer with either the primary antibody targeting phosphorylated‐BCKDH (#A304‐672A‐M 1:1000; Thermo Fisher Scientific), total‐BCKDH (#A303‐790A, Thermo Fisher Scientific), phosphorylated‐AMPK‐threonine172 (#2531–1:1000 v/v; Cell signaling, Leiden, the Netherlands) or αTubulin (T5168‐1:1000 v/v; Sigma‐Aldrich). The next day, blots were washed in TBST and incubated for 1 h in block buffer with the secondary antibody (#7074S‐1:2000 *v*/*v*; Cell Signaling). Blots were washed again and treated with Super Signal West Femto (Thermo Fisher Scientific) to visualize protein bands. Blots were analyzed with a ChemiDoc Touch Imaging system (Bio‐Rad) and band intensities were normalized to tubulin.

### 2.8 Intrahepatic cytokines and chemokines

Intrahepatic concentrations of cytokines (IL‐1b, IL‐4, IL‐6, IL‐10, IL‐15, IL‐17A/F, IL‐33, IFNy, and TNF‐α) and chemokines (MIP1a/CCL3, IP10/CXL10, KC/CXCL1, MIP‐2/CXCL2) were measured in liver biopsies homogenized in lysis buffer as previously reported.^32^ The cytokines and chemokines in the liver were measured using a multiplex immunoassay panel (#KH152AOH; V‐PLEX Custom Mouse Biomarker, Mesoscale Discovery [MSD], Maryland, USA) according to the manufacturer's protocol on a MESO QuickPlex SQ 120 reader (MSD). Total protein concentrations in the same homogenates were assessed with a BCA Protein Assay Kit (Thermo Fisher Scientific, Waltham, MA, USA) to determine the inflammatory factors per mg of protein.

### 2.9 Statistics

Statistical analysis was performed with IBM SPSS statistics version 25.0 (SPSS Inc., Chicago, Illinois, USA). Data were tested for normality with the Shapiro–Wilk test and homoscedasticity with Levene's test (*α* = .05). Normally distributed data with equal variance was tested with a one‐way analysis of variance (ANOVA) with Dunnet's post hoc test using FFD as the control group. Not normally distributed data and/or data with non‐equal variances were tested with the Kruskal–Wallis test and Mann–Whitney post hoc test.

## 3 RESULTS

### 3.1 Valine and isoleucine decreased FFD‐induced hyperinsulinemia and improved circulating liver damage markers independent of effects on body composition

After 38 weeks of FFD, mice developed pronounced obesity with a significantly increased body weight and fat mass and a reduced lean mass, relative to chow (Table 1). Valine or isoleucine did not affect these endpoint readouts. Average caloric intake during the 12‐week intervention period was comparable between FFD and chow, and valine and isoleucine did not affect caloric intake.

**TABLE 1:** Body weight, body composition, and risk factors of metabolic disease

	Chow	FFD	FFD + Val	FFD + Iso
Body weight (g)	34.0 ± 3.7*	46.2 ± 5.0	45.0 ± 5.2	43.4 ± 6.0
Fat mass (% of BW)	11.2 ± 4.3*	37.9 ± 4.2	36.6 ± 6.8	34.7 ± 7.1
Lean mass (% of BW)	85.4 ± 4.1*	59.9 ± 4.1	61.5 ± 5.7	63.8 ± 7.1
12‐week avg food intake (Kcal/mouse/day)	12.6 ± 1.4	13.8 ± 1.0	16.4 ± 2.0	15.0 ± 1.7
Cholesterol (mM)	9.2 ± 1.0*	50.0 ± 10.1	46.3 ± 10.5	42.0 ± 15.9
Triglycerides (mM)	1.6 ± 0.3*	10.3 ± 2.4	8.6 ± 2.6	7.7 ± 4.4
ApoB (mg/ml)	0.4 ± 0.1*	0.7 ± 0.1	0.7 ± 0.1	0.6 ± 0.1*
Glucose (mM)	7.9 ± 0.6*	6.9 ± 0.7	7.6 ± 1.0	7.5 ± 1.3*
Insulin (ng/ml)	0.9 ± 0.3*	4.7 ± 2.0	3.3 ± 1.3*	3.2 ± 1.5*
FD4 (ug/ml)	1.3 ± 0.3	1.7 ± 0.3	1.8 ± 0.6	1.9 ± 1.1
ALT (U/l)	39.6 ± 9.6*	304.8 ± 106.8	251.2 ± 108.7	196.9 ± 90.7*
CK18‐M30 (mU/ml)	219.1 ± 39.6*	383.6 ± 59.2	343.3 ± 42.7	310.5 ± 69.9*

*Note*: Plasma readouts were determined in endpoint plasma (*t* = 38) collected after dietary intervention with valine (FFD + Val) or isoleucine (FFD + Iso) for 12 weeks.

* Asterisk indicates significance of difference compared to the FFD control group with *p* < .05, data shown here as mean ± SD.

**FIGURE 1:**
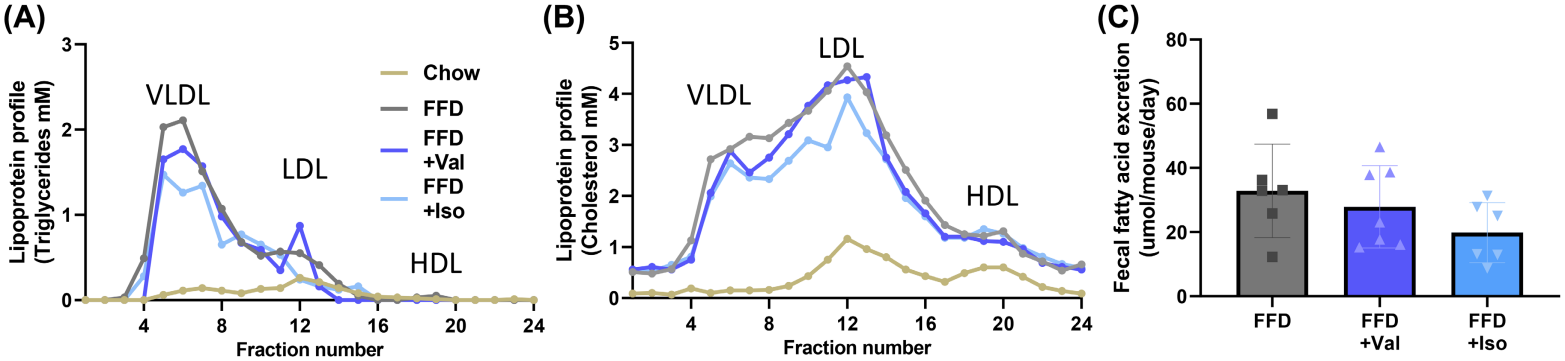
Lipoprotein profiles were analyzed in plasma (pooled from *n* = 15 mice per treatment group) by fast protein liquid chromatography (FPLC). In the obtained fractions, total (A) cholesterol and (B) triglycerides were measured and the results were plotted as profiles. (C) Fecal fatty acid excretion. Data presented as mean ± SD.

FFD feeding induced hyperlipidemia with pronounced increases in plasma cholesterol and TGs, which were non‐significantly lowered by valine and isoleucine. Plasma lipoprotein profiles demonstrated that the FFD‐induced increase in plasma TGs can mainly be ascribed to an increase in VLDL lipoprotein particles and the increase in plasma cholesterol to an increase in (V)LDL‐sized particles (Figure 1A,B). Valine had no marked effect on the lipoprotein profiles, while isoleucine seemed to lower TG in VLDL and cholesterol in VLDL/LDL. Indeed, fasted plasma ApoB mainly present on (V)LDL particles was significantly increased with FFD, while valine did not affect ApoB concentrations and isoleucine significantly lowered ApoB (Table 1).

To analyze whether the BCAAs would affect the uptake of dietary fat, fecal fatty acid concentrations were determined. Treatment with FFD resulted in an average fatty acid excretion of 32.8 μmol/mouse/day. Valine and isoleucine did not affect the excretion of fecal fatty acids showing comparable values (27.8 and 19.8 μmol/mouse/day, respectively) (Figure 1C).

Fasting glucose was lower in the FFD group compared to chow, and valine reversed this FFD effect non‐significantly whereas this reversal reached significance in the isoleucine group (Table 1). FFD strongly induced hyperinsulinemia compared to chow‐fed mice. Valine and Isoleucine significantly reduced fasting insulin concentrations compared to FFD, indicative of a BCAA‐induced improvement in insulin resistance.

Gut permeability, assessed by analysis of orally administered FD4 and subsequent uptake in plasma, was non‐significantly elevated with FFD relative to chow, and not affected by either of the BCAAs (Table 1).

FFD and chow feeding groups demonstrated comparable BCAA concentrations in fasting plasma. Consistent with the dietary supplementation of valine and isoleucine, a few mice of the respective treatment groups showed modest elevations of these BCAA an effect which became significant for isoleucine. Leucine concentrations were comparable in all groups (Figure S1).

Circulating markers of liver injury ALT and CK18‐M30 were significantly elevated with FFD relative to chow (Table 1). Valine tended to lower these markers. Isoleucine had a more pronounced lowering effect and significantly decreased ALT and CK18‐M30. Together these biomarker analyses indicate potential hepatoprotective effects of valine and isoleucine independent of effects on body composition.

### 3.2 Valine and isoleucine upregulate BCAA metabolism genes

We next evaluated whether BCAA treatment affected the hepatic gene expression program that controls BCAA metabolism using NGS.

**FIGURE 2:**
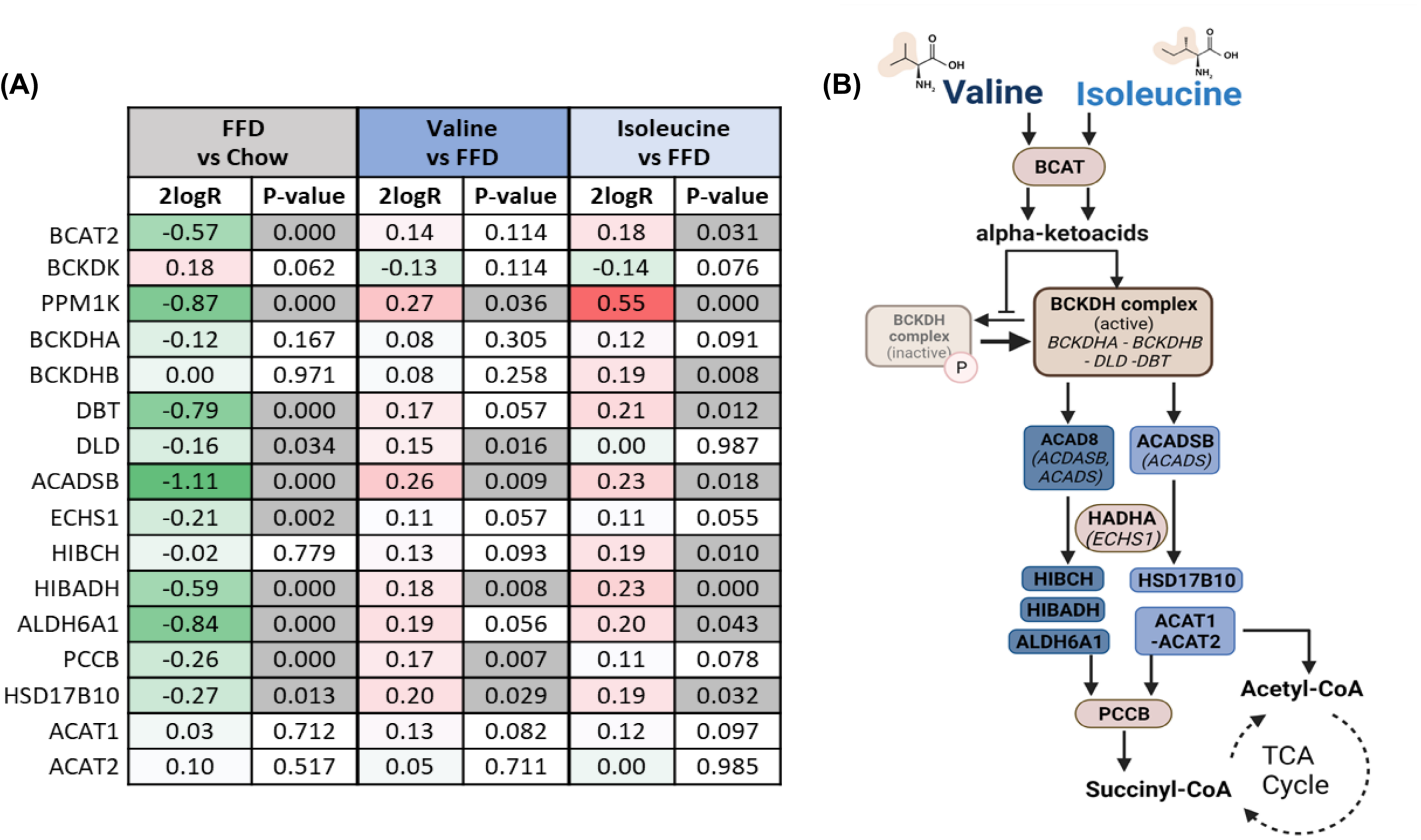
Changes in expression of genes involved in BCAA metabolism. (A) Gene expression changes are expressed in 2log fold‐change (2logR), red indicates upregulation in gene expression, green indicates downregulation and *p*‐values indicate significant changes marked in gray. (B) Graphical overview of valine and isoleucine catabolism. Valine and isoleucine can promote their own breakdown via the branched‐chain α‐ketoacid dehydrogenase (BCKDH) complex. Valine can further be metabolized into succinyl‐CoA while isoleucine provides acetyl‐CoA or succinyl‐CoA, which can enter the tricarboxylic acid (TCA) cycle.

FFD strongly decreased the hepatic mRNA expression of BCAT and genes that encode for the BCKDH complex (BCKDHA, BCKDHB, DBT, DLD), and many related genes (PPM1K, ACADSB, ECHS1, HIBADH, ALDH6A1; Figure 2A) that encode for proteins required for BCAA catabolism (graphical overview in Figure 2B). Valine, and to an even greater extent, isoleucine reversed the FFD‐induced effects resulting in the upregulation of many of these genes (Figure 2A). Taken together, gene expression data indicate that treatment with valine and isoleucine stimulates genes involved in the catabolism of BCAAs in the liver, thereby counteracting the effect of FFD feeding during NASH development. Hepatic protein expression analysis demonstrated that FFD significantly increased the ratio between phosphorylated‐BCKDH and total‐BCKDH, yet valine and isoleucine did not significantly lower this ratio (Figure S2).

### 3.3 Valine and isoleucine reduced hepatic lipid accumulation associated with an improvement in lipid metabolism

**FIGURE 3:**
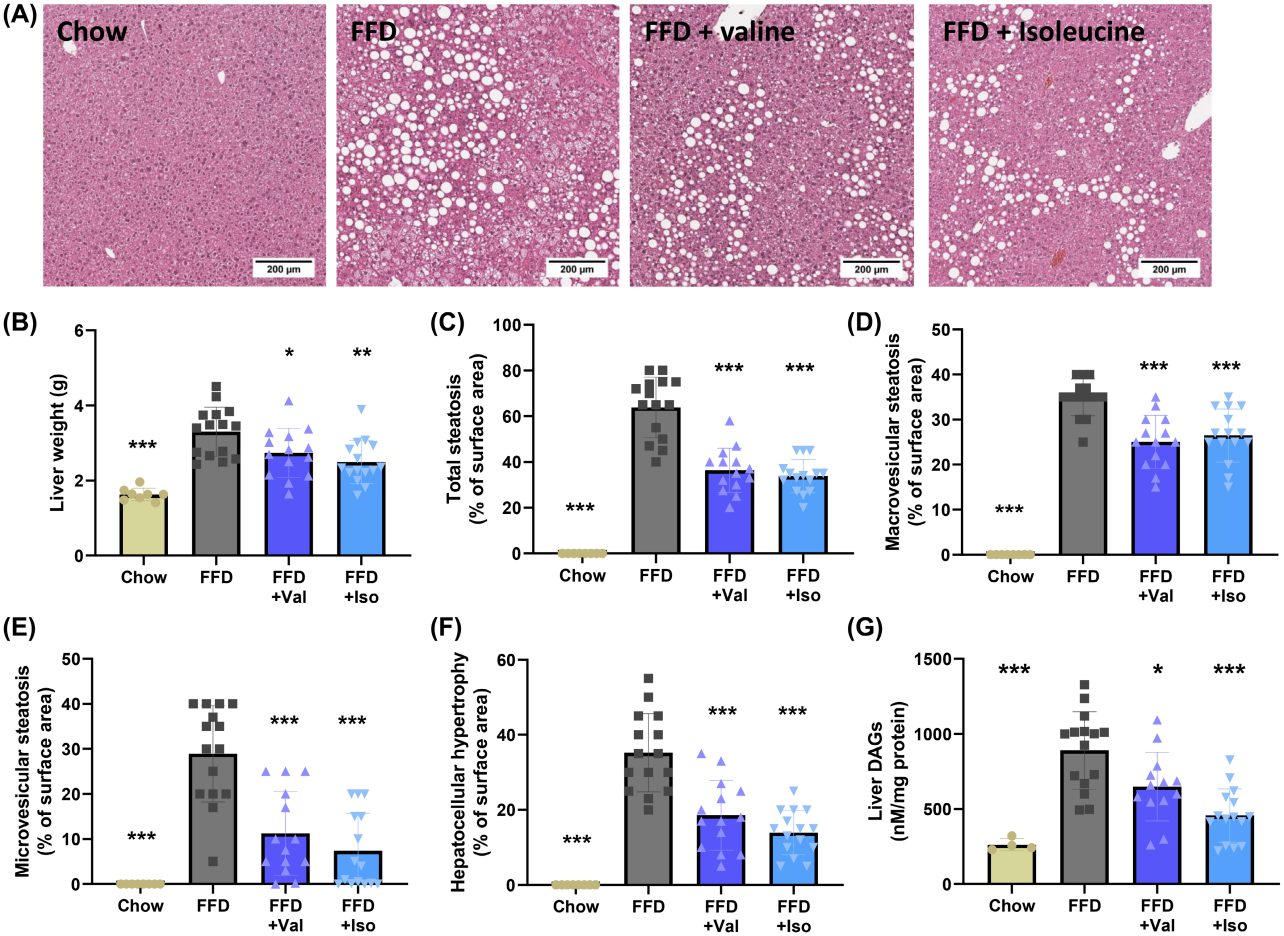
(A) Representative images of hematoxylin and eosin‐ (H&E) stained liver cross‐sections, (B) liver weight, histological analysis of the HE‐stained liver cross‐section for: (C) total steatosis, (D) macrovesicular steatosis, (E) microvesicular steatosis, (F) hepatocellular hypertrophy. (G) intrahepatic diacylglycerol (DAG) lipids expressed per mg protein. Data are presented as mean ± SD, *p < .05 or **p < .01 or ***p < .001 compared to the FFD control group.

To investigate the potential hepatoprotective effects of valine and isoleucine on the tissue level, we examined liver weight and histopathological features of NASH. FFD feeding increased liver weight and induced pronounced steatosis with comparable induction of macrovesicular and microvesicular steatosis and increased hepatocellular hypertrophy (Figure 3A–F). Valine and isoleucine‐treated livers were lower in weight, showing a reduction of 15% and 25%, respectively (Figure 3B). Macrovesicular steatosis was decreased by 28% with valine and by 24% with isoleucine (Figure 3C,D). The decrease in microvesicular steatosis was even more pronounced, with marked reductions by 61% (valine) and 71% (isoleucine) relative to FFD (Figure 3E). Hepatocellular hypertrophy was lowered by 47% and 60% with valine and isoleucine, respectively (Figure 3F).

Consistent with the histological steatosis effects, FFD markedly increased intrahepatic diacylglycerols (DAGs) (Figure 3G), a lipid that blocks insulin action at the level of its receptor.^7^ The FFD‐induced accumulation was significantly suppressed by both valine and isoleucine, providing a mechanistic rationale for the significant reduction of hyperinsulinemia observed with both BCAAs.

The pronounced effects on liver histology were substantiated by NGS and subsequent upstream regulator analyses (IPA bioinformatics tool^31^). The IPA analysis predicts the activation state of an upstream regulator (e.g., transcription factor, metabolic enzyme, signaling cytokine) based on the expression pattern of (target) genes downstream from this regulator (Table 2). FFD affected lipid metabolism in multiple ways and several regulators of lipid synthesis (e.g., SREBF1) and anabolic processes (e.g., IGF1, AGT), were activated, whereas regulators involved in energy homeostasis and lipid catabolism (e.g., AMPK, ACOX1, EHHADH), as well as mitochondrial biogenesis (e.g., PPARGC1A, CLUH) and energy dissipation (e.g., UCP1), were deactivated. In line with this FFD lowered pThr172‐AMPK with borderline significance (*p* = .06) (Figure S3). Valine and isoleucine counter‐regulated many of these FFD gene expression effects (Table 2), which was further supported by canonical pathway analysis. For example, valine and isoleucine stimulated the canonical triglyceride degradation pathway (*Z* = 2.0; *p* < .05 and *Z* = 3.0; *p* < .05, respectively), and especially isoleucine affected the canonical fatty acid β‐oxidation pathway (*Z* = 2.2; *p* < .05) and canonical AMPK signaling (*Z* = 1.9; *p* < .05). Taken together, valine and isoleucine supplementation inhibits lipid synthesis pathways and stimulates hepatic lipid catabolism pathways in line with the observed reduction of liver steatosis.

**TABLE 2:**
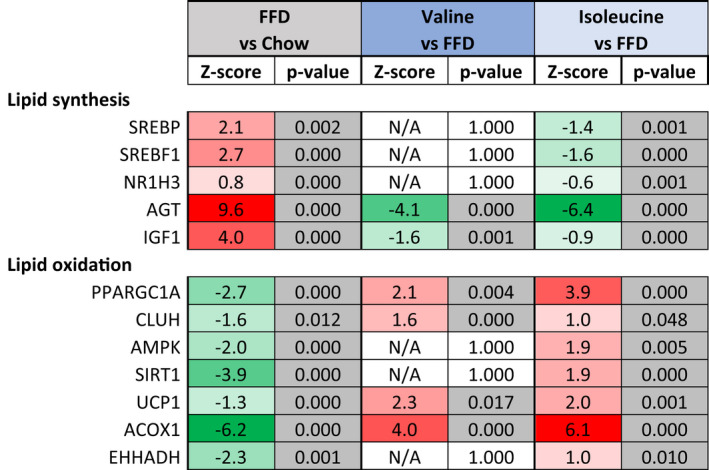
Upstream regulator analysis of key lipid metabolism regulators

*Note*: The activity of an upstream regulator was calculated based on gene expression changes of all downstream target genes. A negative Z‐scoreindicates inhibition of the respective regulator or pathway (green color) and a positive Z‐score indicates activation (red color). The p‐value <.05 in gray indicates significant enrichment of the target genes downstream of a regulator, i.e., that more downstream genes are affected than can be expected by chance. N/A indicates an insufficient number of differentially expressed genes to predict the activation state of an upstream regulator.

### 3.4 Valine and isoleucine attenuate hepatic lipid peroxidation, a hallmark of oxidative stress

**FIGURE 4:**
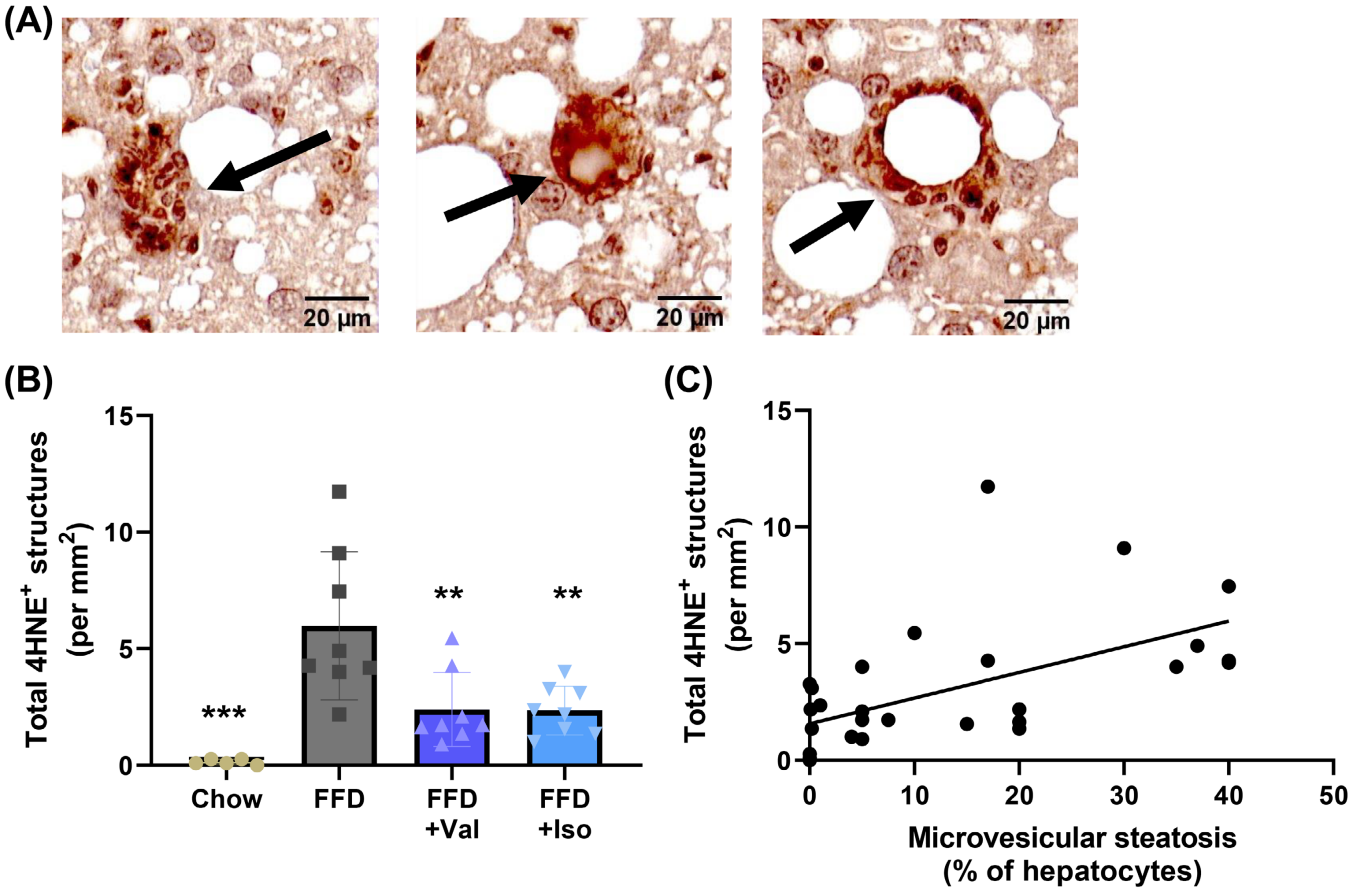
The oxidative‐stress‐induced lipid peroxidation marker 4‐hydroxynonenal (4‐HNE) was analyzed in liver cross‐sections using immunohistochemistry. (A) Representative photomicrographs of structures with 4‐HNE‐positive immunoreactivity. (B) Quantification of the 4‐HNE‐positive structures (C). Correlation of 4‐HNE‐positive structures with microvesicular steatosis. Data are presented as mean ± SD, *p < .05 or **p < .01 or ***p < .001 compared to the FFD control group.

The pronounced effect of BCAA on microvesicular steatosis, a form of steatosis that is associated with mitochondrial dysfunction,^8^ prompted us to examine oxidative stress in the liver as mitochondria are the main producers of reactive oxygen species. 4‐hydroxynonenal (4‐HNE) is a marker for lipid peroxidation induced by oxidative stress, and 4‐HNE‐positive immunoreactivity (IR) was analyzed in the liver by counting the number of 4‐HNE‐positive structures (typical examples shown in Figure 4A). Notably, closer inspection revealed that 4HNE‐positive immunoreactivity was also present in enlarged hepatocytes (as exemplified in Figure S4). This observation is consistent with the view that oxidative stress associated with 4HNE formation is a pathophysiological hallmark already observable in hepatocytes prior to cell death and inflammation. 4‐HNE‐positive IR was practically absent in chow and FFD feeding strongly increased the 4‐HNE‐positive IR (Figure 4B). This FFD‐induced increase was significantly suppressed with both valine and isoleucine. Interestingly, the number of 4‐HNE positive structures also significantly correlated with microvesicular steatosis (Pearson correlation; *R* = .57, *p* = .0012) (Figure 4C).

### 3.5 Valine and isoleucine attenuate hepatic inflammation

**FIGURE 5:**
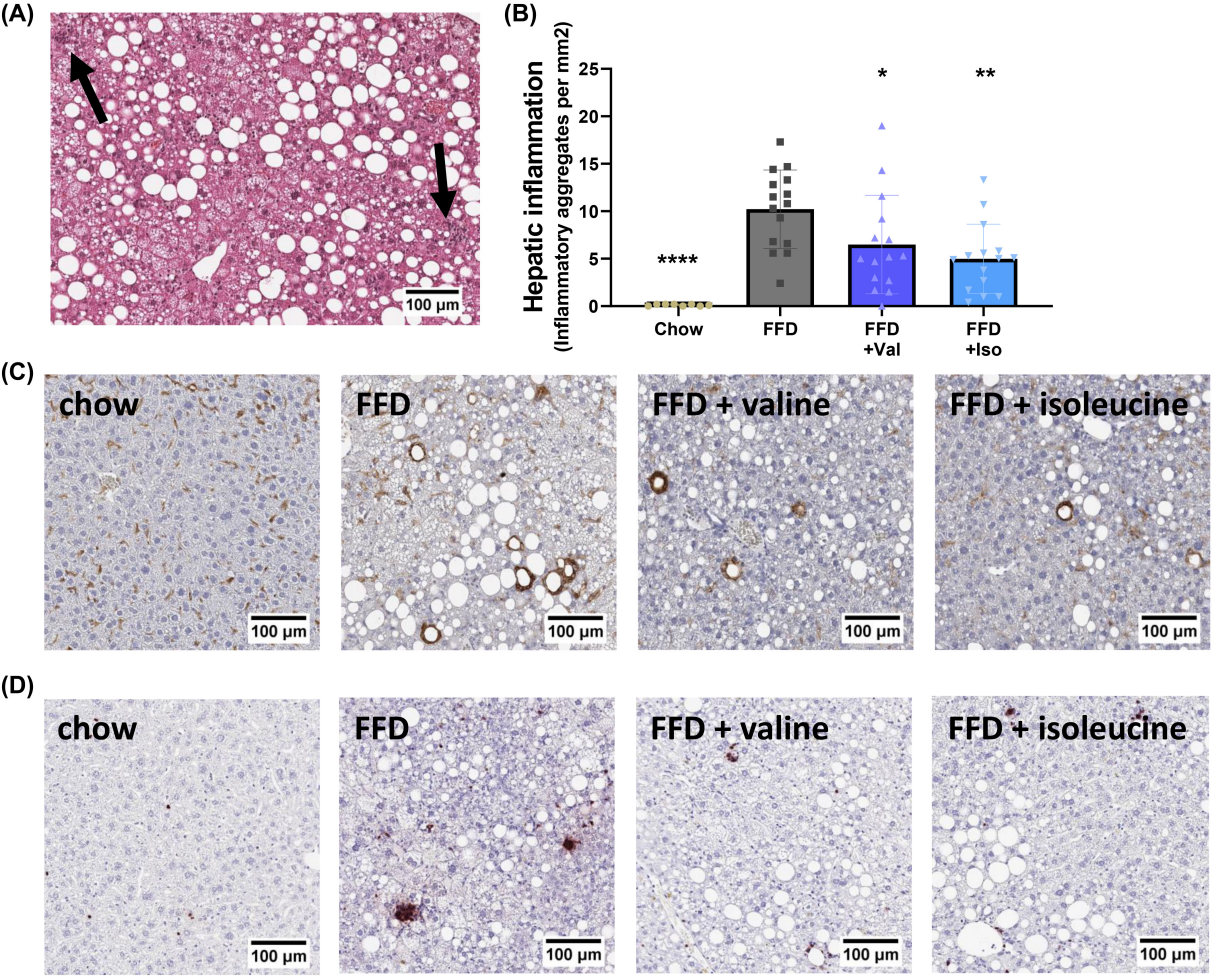
Hepatic inflammation. (A) Representative image of hepatic inflammation in hematoxylin and eosin (H&E) stained liver cross‐sections, quantified by counting (B) the number of inflammatory aggregates and expressed per mm^2^. Representative photomicrographs of (C) F4/80 immunoreactivity and (D) GR‐1 immunoreactivity in the different treatment groups. Data are mean ± SD, *p < .05 or **p < .01 or ***p < .001 compared to the FFD control group.

Hepatic inflammation was histologically analyzed (Figure 5A–D) and complemented by gene expression analysis of inflammatory pathways using NGS datasets.

Liver inflammation expressed as the number of inflammatory aggregates per mm^2^ was practically absent in the chow‐fed animals, whereas FFD‐mice developed pronounced hepatic inflammation. Valine suppressed the number of inflammatory aggregates by 36% and isoleucine even by 51% (Figure 5B). The histological analysis of lobular inflammation was supported by immunohistochemical staining of F4/80, a macrophage marker, (Figure 5C), and GR‐1, a neutrophil marker (Figure 5D). F4/80 and GR‐1 immunoreactivity was strongly increased by FFD feeding and less pronounced in the groups treated with valine and isoleucine.

Consistent with this marked suppression of lobular inflammation, upstream regulator analysis indicated that FFD‐feeding resulted in a pronounced activation of several proinflammatory cytokine pathways, including the signaling cascades downstream of IL‐1β, CSF1, and TNF‐α (Table 3). FFD also activated chemokine signaling implicated in NASH development among which the inflammatory pathways controlled by CCL2, CCR2, and CXCL12. Both valine and isoleucine deactivated specific proinflammatory signaling pathways, including IL‐1β, TNF‐α, CCR2, and CXCL12. The anti‐inflammatory effects of isoleucine were often more pronounced and included suppression of IL17A signaling which was not affected by valine. Subsequent analysis of canonical pathways associated with inflammation revealed that FFD activated NF‐κB signaling (*Z* = 2.4; *p* < .05), chemokine signaling (*Z* = 3.0; *p* < .05), MIF regulation of innate immunity (*Z* = 3.2; *p* < .05), IL‐8/KC signaling (*Z* = 4.7, *p* < .05) and fMLP signaling in neutrophils (*Z* = 3.4 and *p* < .05). The observed downregulation of CXCL12 with both BCAAs indicates an attenuation of neutrophil chemotaxis. Indeed, valine and isoleucine decreased IL‐8/KC signaling (both with *Z* = −2.5 and *p* < .05) and non‐significantly lowered fMLP signaling in neutrophils.

**TABLE 3:**
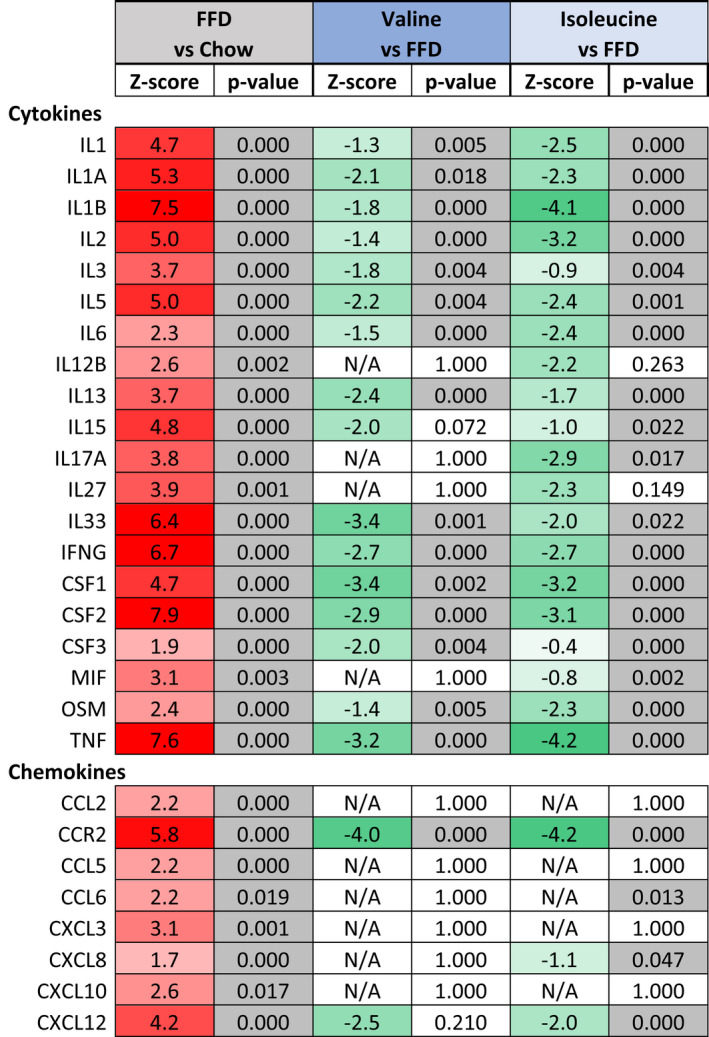
Upstream regulator analysis of inflammatory regulators

*Note*: The activity of an upstream regulator was calculated based on gene expression changes of all downstream target genes. A negative Z‐score indicates inhibition of the respective regulator or pathway (green color) and a positive Z‐score indicates activation (red color). The p‐value <.05 in gray indicates significant enrichment of the target genes downstream of a regulator, i.e., that more downstream genes are affected than can be expected by chance. N/A indicates an insufficient number of differentially expressed genes to predict the activation state of an upstream regulator.

Consistent with the increase in liver inflammation with FFD on histological and transcriptomics levels, FFD increased the intrahepatic protein concentrations of pro‐inflammatory cytokines IL‐1β, IL‐6, IL‐15, IL‐17A/F, IL‐33, and TNF‐α and chemokines KC/CXCL1, MIP‐2/CXCL2, and IP10/CXL10. At the same time, FFD also increased cytokines IL‐4 and IL‐10 with anti‐inflammatory properties. Isoleucine but not valine significantly attenuated the expression of the pro‐inflammatory chemokines MCP1/CCL2, KC/CXCL1, and MIP‐2/CXCL2. In addition, valine and isoleucine further increased IL‐4 concentrations indicating a hepatoprotective effect of both BCAA (Table 4).

**TABLE 4:** Intrahepatic cytokines and chemokines

	Chow	FFD	FFD + Val	FFD + Iso
IL‐1β	4.2 ± 1.1*	16.9 ± 4.3	19.5 ± 11.1	13.9 ± 7.7
IL‐4	0.02 ± 0.01*	0.07 ± 0.02	0.12 ± 0.03*	0.11 ± 0.04*
IL‐6	3.5 ± 0.7*	6.5 ± 0.9	6.6 ± 1.1	6.6 ± 1.4
IL‐10	0.4 ± 0.1*	1.0 ± 0.3	1.0 ± 0.2	1.2 ± 0.4
IL‐15	5.2 ± 1.9*	15.0 ± 3.7	12.8 ± 2.5	15.2 ± 4.0
IL17A/F	0.2 ± 0.1*	0.4 ± 0.0	0.3 ± 0.0	0.4 ± 0.1
IL‐33	6.7 ± 0.9*	20.7 ± 7.1	23.5 ± 10.4	22.9 ± 11.3
TNF‐α	0.2 ± 0.1*	0.9 ± 0.2	1.3 ± 0.7	0.9 ± 0.4
IFNγ	0.0 ± 0.0	0.1 ± 0.0	0.1 ± 0.1	0.1 ± 0.0
MCP‐1/CCL2	1.5 ± 0.6*	12.8 ± 2.9	13.5 ± 3.4	9.5 ± 4.2*
MIP‐1a/CCL3	3.5 ± 3.7	13.0 ± 7.2	14.6 ± 15.0	11.5 ± 10.1
KC/CXCL1	1.6 ± 0.3*	6.1 ± 1.2	6.2 ± 2.0	4.3 ± 1.2*
MIP‐2/CXCL2	0.6 ± 0.4*	5.5 ± 2.4	4.8 ± 2.6	3.0 ± 1.1*
IP‐10/CXCL10	4.7 ± 1.2*	15.8 ± 4.2	18.3 ± 8.1	14.1 ± 5.4

*Note*: The concentrations of cytokines and chemokines are expressed in pg per mg protein, determined in liver homogenates after dietary intervention with valine (FFD + Val) or isoleucine (FFD + Iso) for 12 weeks.

* Asterisk indicates significance of difference compared to the FFD control group with *p* < .05, data shown here as mean ± SD.

## 4 DISCUSSION

The objective of this study was to investigate the potential hepatoprotective effects of valine and isoleucine individually and to study whether these BCAAs can attenuate NASH development when administered therapeutically, i.e., starting treatment when features of the metabolic syndrome and NASH are already established. NGS was used to gain insight into the comprehensive effects of the BCAAs on canonical pathways critical for liver metabolism and the activation state of upstream regulators, which exceeds conventional mRNA expression analysis. Supplementation of FFD with valine or isoleucine reduced liver steatosis and associated oxidative stress as demonstrated by an attenuation of the lipid peroxidation marker 4‐HNE. Both BCAAs restored the detrimental effects of FFD feeding on hepatic metabolic homeostasis: they corrected the expression of BCAA metabolism genes and counter‐regulated FFD effects on key regulators involved in energy homeostasis and lipid metabolism (e.g., AMPK, SREBPF1). These beneficial metabolic effects were accompanied by a pronounced reduction of immune cell‐mediated liver inflammation. These cellular effects were further substantiated by the suppression of critical inflammatory pathways (e.g., IL‐1β and TNF‐α signaling) known to be upregulated in NASH patients.^18^

The observed hepatoprotective effects of valine and isoleucine were independent of changes in body weight or body composition and gut permeability, consistent with previous reports demonstrating no effect of BCAA on body weight development in obesity.^14^, ^33^ Cardiovascular risk factors such as plasma total cholesterol and triglycerides were non‐significantly lowered with valine and isoleucine, in line with a slightly lower TG in VLDL and cholesterol in VLDL/LDL in the lipoprotein profiles. These results suggest that the observed reduction of liver steatosis is not due to an enhanced hepatic lipid output, i.e., enhanced assembly and secretion of VLDL particles from the liver into the circulation, as the lipid output was in fact lowered by the BCAAs. These observations are in line with an in vitro study which demonstrated that BCAAs had minor effects on apoB (primary protein on VLDL and LDL lipoprotein particles) secretion by hepatocytes.^34^ In addition, we showed that fecal fatty acid output was comparable between groups indicating that the decrease in liver steatosis can also not be explained by an enhanced fatty acid excretion via the feces, implying an effect on hepatic lipid handling and utilization.

Interestingly, the microvesicular form of steatosis was most strongly decreased with valine and isoleucine. The development of microvesicular steatosis is related to mitochondrial dysfunction and impaired FA oxidation, which in turn promotes the accumulation of hepatic FA in small lipid droplets.^11^ The observed decrease in microvesicular steatosis therefore suggests an improvement in lipid handling related to mitochondrial function.^35^, ^36^ Interestingly, we found that valine and isoleucine promoted mitochondrial biogenesis as both PPARGC1α^37^ and CLUH,^38^ which were suppressed by FFD were reactivated with the BCAAs. In line with these data, also hepatic lipid metabolism appears to be stimulated with valine as well as isoleucine: key metabolic regulators involved in lipid oxidation (ACOX1, EHHADH) and energy homeostasis (AMPK) were activated, and key regulators involved in fatty acid synthesis (SREBF1) and anabolic processes (IGF1, AGT) were conversely inactivated. The NGS upstream regulator effects on AMPK were more pronounced than the Western blot analyses, i.e., a trend decrease in AMPK‐phospho‐Threonine 172 and no effect with the BCAAs. However, in contrast to the Western blot analysis, an upstream regulator analysis uses gene expression data of *all known* downstream target genes of AMPK to predict its state of activation or deactivation. The NGS analysis thus indirectly involves all possible protein modifications, including potential AMPK deactivations regulated by diacylglycerols^39^, ^40^ and inflammation,^12^ and thus not limited to a particular type of protein modification (e.g., phosphorylation, acetylation, allosteric inhibition/activation). The lipids in the smaller lipid droplets, which manifest as microvesicular steatosis, are thought to be easier metabolizable than large droplets.^41^ When lipids are used to meet the energy demands of the liver smaller lipid droplets are thought to be metabolized first, which decreases the likelihood that larger intracellular lipid droplets (macrovesicular steatosis) are being formed.^41^ Macrovesicular steatosis impairs hepatocellular function and activates stellate cells,^42^ which is supported by the correlation between macrovesicular steatosis and fibrosis in rodent models of disease.^43^

Moreover, oxidative stress has been linked to the manifestation of microvesicular steatosis^8^ and increased lipid peroxidation from ROS species with the formation of 4‐HNE adducts in liver.^16^, ^28^ Mitochondria are among the main producers of ROS, which increase when mitochondria become dysfunctional, as has been reported in NASH patients.^44^ The observed decrease in 4‐HNE immunoreactivity in the valine and isoleucine treated groups indicates less oxidative stress and less hepatic lipid peroxidation. This observation could be a consequence of better functioning mitochondria and/or greater numbers of mitochondria because valine and isoleucine activated PPARGC1A and CLUH which control critical steps in mitochondrial biogenesis.^37^, ^38^ This study also confirmed the presumed positive association between oxidative stress and microvesicular steatosis showing a significant correlation between 4‐HNE immunoreactivity and microvesicular steatosis. Our results are also in line with a recent study that demonstrated that a mixture of BCAA prevented fat accumulation and mitochondrial dysfunction in hepatocytes of alcohol‐consuming rodents.^45^ These protective effects on mitochondria appear to be specific for BCAAs as shown in a rodent study comparing a mixture of BCAAs to a mixture of amino acids from casein.^45^

Besides improving mitochondrial functioning, valine and isoleucine may alleviate the accumulation of lipids in the liver via their effect on insulin. Fasting insulin was strongly increased by FFD feeding and this induction was significantly lowered with both BCAAs. Moreover, insulin promotes SREBP1‐mediated de novo lipogenesis^4^, ^5^ in the liver and upstream regulator analysis indeed demonstrated that FFD feeding strongly enhanced SREBP1 activity. By contrast, valine and isoleucine lowered circulating plasma insulin and deactivated SREBP1. This effect on insulin and SREBP1 may contribute to the reduction of steatosis. Besides these direct effects in the liver, insulin also has extrahepatic effects on lipases and in the adipose tissue.^7^ Improved insulin action can lower fat fluxes from the adipose tissue to the liver which also reduces metabolic overload and fat accumulation in the liver, including intrahepatic DAGs which can build up when fatty acid fluxes become too high or fatty acid catabolism is impaired. In the present study, FFD treatment indeed resulted in a significant increase in liver DAGs, and this FFD effect was counter‐regulated by valine and isoleucine.

DAGs are causal to insulin resistance because DAGs impair insulin signaling at the level of its receptor via recruitment of novel PKCs (in the liver and other organs including skeletal muscle and WAT).^46^ Importantly, also in humans it has been shown that DAGs correlate with insulin sensitivity measured by clamp technique.^46^ Hence, it is amenable that intercepting with BCAA adjusts the overall metabolism and thereby reduces the build‐up of DAGs and associated insulin resistance, a key driver of NAFLD development.

AMPK is one of the major regulators of energy homeostasis and activated AMPK inhibits fatty acid synthesis (by phosphorylating and inhibiting SREBP1)^47^ at the same time stimulating β‐oxidation (e.g., directly via PCG1a and indirectly via ACOX1).^48^ Furthermore, AMPK is a ROS and nutrient sensor and also controlled by diacylglycerols and inflammatory signaling pathways (TNF‐α /TBK1), which were increased and activated by FFD in the present study and suppressed by valine and isoleucine on the pathway level. In essence, the observed attenuation of inflammation with valine and isoleucine and the improvement of lipid metabolism should not be interpreted as separate observations. Because metabolism and inflammation are evolutionarily interconnected via enzymes such as AMPK and transcriptional regulators such as peroxisome proliferator‐activated receptors (PPARs),^12^, ^49^ it is likely that an improvement of metabolic processes by BCAAs attenuated the induction of inflammatory pathways and, vice versa. Quenching of inflammatory cytokine or chemokine signaling cascades with valine or isoleucine allowed metabolism to function closer to optimal homeostasis. It is difficult to disentangle metabolic processes and low‐grade chronic inflammation during the development of NAFLD/NASH. Mitochondria with their primary function in metabolism could be implicated in the initiation of chronic inflammation, in particular, once they disintegrate together with the cells in which they reside. Mitochondrial proteins contain n‐formyl‐met‐leu‐pheu (fMLP) peptides which exert chemotactic function for neutrophils once released from necrotic cells.^50^ It has been demonstrated that human mitochondrial formyl‐peptides promoted chemotaxis of immune cells and oxidative burst of neutrophils in vitro.^51^ Interestingly, FFD feeding activated the canonical pathways relevant for neutrophil recruitment (e.g., IL‐8/KC and fMLP signaling) and valine and isoleucine attenuated these canonical pathways, consistent with a decrease in hepatic immunoreactivity of GR‐1 with valine and isoleucine, and indicative of decreased neutrophil infiltration. This observation is noteworthy because neutrophils are components of the inflammatory aggregates that were reduced with valine and isoleucine, and they constitute a hallmark of human NASH and drivers of the pathogenesis.^52^, ^53^ In line with histology and the NGS‐based predictions, KC/CXCL1 protein levels were strongly increased by FFD and reduced with isoleucine. At this point, we would like to emphasize that the NGS‐based prediction of an entire proinflammatory cytokine/chemokine signaling cascade may deviate from the protein expression level of the respective cytokine/chemokine. The reason is that the NGS approach predicts the signaling cascade on basis of dozens of downstream target genes of a cytokine/chemokine whereas the protein concentration of this cytokine/chemokine per se does not inform the activity of its downstream signaling cascade.

Published rodent studies investigating the effect of BCAA restriction and BCAA supplementation may seem contradictory because one might expect opposite effects. In general, both strategies can improve metabolic processes depending on the type and stage of dysmetabolism at the start of the treatment and how metabolic disturbances are induced. Published studies vary strongly regarding diets and their composition, the study design (prevention of diseases versus intervention in already deranged metabolism), the condition under which tissues were collected (feeding vs fasting state), and the readouts.^14^, ^22^, ^54^, ^55^, ^56^, ^57^, ^58^, ^59^, ^60^, ^61^, ^62^, ^63^, ^64^, ^65^, ^66^, ^67^, ^68^, ^69^, ^70^, ^71^, ^72^, ^73^ BCAA restriction is not necessarily the opposite of supplementing a high caloric diet with BCAAs, because BCAA restriction can body weight^70^, ^71^, ^72^, ^73^ which was not observed in the present study. In BCAA restriction experiments, White et al.^72^ Fontana et al.^73^ and Yu et al.^71^ reported improvements in metabolic health readouts including improvements in hepatic insulin sensitivity and energy expenditure. However, BCAA restricted animals in these studies were significantly lower in weight compared to their respective controls and health effects may thus be a consequence of weight loss.

In the present study, we examined the effect of valine and isoleucine in FFD‐pretreated mice that were in a particular dysmetabolic state at the start of the intervention, i.e., obese mice with associated dyslipidemia and hyperinsulinemia^16^ exhibiting elevated diacylglycerol concentrations at tissue level which we and others^74^, ^75^, ^76^ consider to be critical for the development of insulin resistance. Herein, BCAA affected the accumulation of DAGs at the tissue level and improve insulin resistance assessed by HOMA. In Ldlr−/−.Leiden mice, DAGs accumulate specifically in response to FFD feeding but not in mice fed chow diet despite comparable calorie intake. The accumulation is thus very likely a consequence of both higher fatty acid fluxes from chronically inflamed WAT depots and a higher intake of calories from fat because the total caloric intake was comparable among the groups. It is possible that under experimental studies in which DAGs do not accumulate (e.g., chow feeding in general), effects on insulin resistance cannot be found which may explain at least some of the discordant observations. For instance, a study by White et al. demonstrated that BCAA supplementation in healthy chow‐fed rats did not induce insulin resistance,^72^, ^77^ possibly because DAGs are not increased (neither by chow nor by the BCAA themselves). Rising circulating BCAA concentrations via the drinking water in high fat/high sucrose or high‐fat diet‐treated mice did not modulate insulin resistance in obese mice.^59^ This observation differs from our finding which could be related to the differences regarding the administration route (via drinking water versus dietary ad mix), differences in doses and treatment time, and alternative DAG‐independent mechanisms. For example, BCAAs can activate mTOR/p70S6 kinase and potentially affect insulin receptor phosphorylation.^78^ In general, it can be said that lipid‐induced insulin resistance is predominantly mediated by DAGs, in addition to this lipid‐mediated mechanism also chronic tissue inflammation (e.g., cytokines and chemokines) can impair insulin signaling.^74^, ^75^, ^76^ In many published high fat diet‐induced obesity studies, information about DAG concentrations on the tissue level is not available, and it is, therefore, difficult to provide an overall explanation for discrepancies between studies.

A limitation of the current study is that we did not sacrifice a group of mice at 26 weeks, i.e., the time point at which interventions were started, to assess the level of NASH and liver fibrosis prior to treatment which would have allowed us to assess a putative regression of histological features. Therefore, we cannot differentiate whether valine and isoleucine merely inhibited further NASH progression from 26 weeks onwards or whether they were even able to regress the disease. Based on extensive time course studies in the same mouse strain (same age, same sex, same diets) the expected level of total steatosis at 26 weeks is 50%–60% as reported.^16^ This then would point to a regression of the disease with valine and isoleucine within 12 weeks of treatment because both treatment arms displayed only about 34% total steatosis. The sacrifice conditions (5‐h fasting in the present experiment) were probably not optimal to determine circulating BCAA because dietary BCAA seemed to be rapidly metabolized and only a few mice exhibited elevated levels in the fasting state. Similarly, fasting conditions may have compromised the assessment of protein modifications with a short half‐life such as the phosphorylation status of BCKDH. More comprehensive analyses that rely on counts of mRNA transcripts with longer half‐life and that involve dozens of genes of a pathway or signaling cascade may therefore be more accurate under our sacrifice circumstances. Leucine was not included in this study because leucine reportedly reduces food intake,^79^ therefore, it would not have been possible to dissociate specific metabolic effects of leucine from its effects on food intake given the diet‐induced nature of the model used herein. Another limitation of the study is the absence of metabolic flux measurements, which were beyond the initial scope.

**FIGURE 6:**
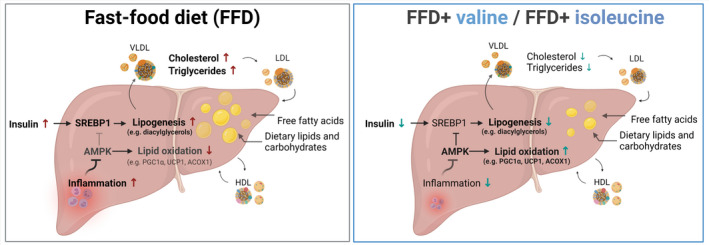
Overview of the different mechanisms which promote NASH development during FFD feeding and were corrected with valine or isoleucine supplementation, including multiple lipid metabolism processes resulting in reduced NASH‐associated steatosis and inflammation.

In conclusion, intervention with dietary valine or isoleucine attenuated already established obesity and insulin resistance‐associated NASH. The BCAAs do not affect a specific disease pathway but affect multiple metabolic and inflammatory processes simultaneously as summarized in Figure 6. The marked decrease in microvesicular steatosis may at least partly be explained by improved lipid processing and oxidation in the liver and concomitant suppression of de novo lipid synthesis as determined indirectly by NGS on the pathway level. An improved catabolism of hepatic lipids is further supported by a slight reduction in apoB and lipoproteins (reflecting lower hepatic lipid output) as well as the absence of fatty acid excretion via the feces. Improvement of hepatic lipid metabolism is also supported by the pronounced reduction in plasma insulin, hepatic diacylglycerols, lipid peroxidation, and lobular inflammation, the latter being substantiated by a profound suppression of critical inflammatory proteins and pathways. Altogether this study demonstrates that valine and isoleucine constitute nutritional treatments that can help restore metabolic and inflammatory homeostasis in steatotic livers and that such dietary strategies could be combined with pharmaceutical interventions directed at fibrosis.

## AUTHOR CONTRIBUTIONS

Eveline Gart, Kanita Salic, Martine C. Morrison, and Robert Kleemann contributed to the design and conception of the study. Eveline Gart, Wim van Duyvenvoorde, Martien P. M. Caspers, Nikki van Trigt, Jessica Snabel, and Aswin Menke performed the research and acquired the data. Eveline Gart, Kanita Salic, Martine C. Morrison, Jaap Keijer, and Robert Kleemann contributed to the analyses and interpretation of the data. All authors were involved in drafting and revising the manuscript and approved the final version before submission.

## DISCLOSURES

The authors declare no conflicts of interest.

## FUNDING INFORMATION

This study was performed within the public‐private partnership (PPP) ProLiver, a collaboration project that is co‐funded by a PPP Allowance made available by Health~Holland, Top Sector Life Sciences & Health, to stimulate public‐private partnerships. The work described here was also supported by the TNO Research Programs Food and Nutrition, and Biomedical Health (PMC 9 and PMC 13) and assays developed in the ERP Body brain interactions.

## Supporting information


Supplemental figures and caption

Supplemental captions

## Data Availability

The transcriptomics data are available on Gene Expression Omnibus (GEO), data set GSE195798.
