# Supplementary material for: Intervention with isoleucine or valine corrects hyperinsulinemia and reduces intrahepatic diacylglycerols, liver steatosis, and inflammation in Ldlr−/−.Leiden mice with manifest obesity‐associated NASH
Source: FASEB J. 2022 Jul 13;36(8):e22435. doi: 10.1096/fj.202200111R (PMC12166278; doi:10.1096/fj.202200111R)
Supplement: Supplementary file 1 — Supplemental figures and caption [file FSB2-36-e22435-s001.docx]

**
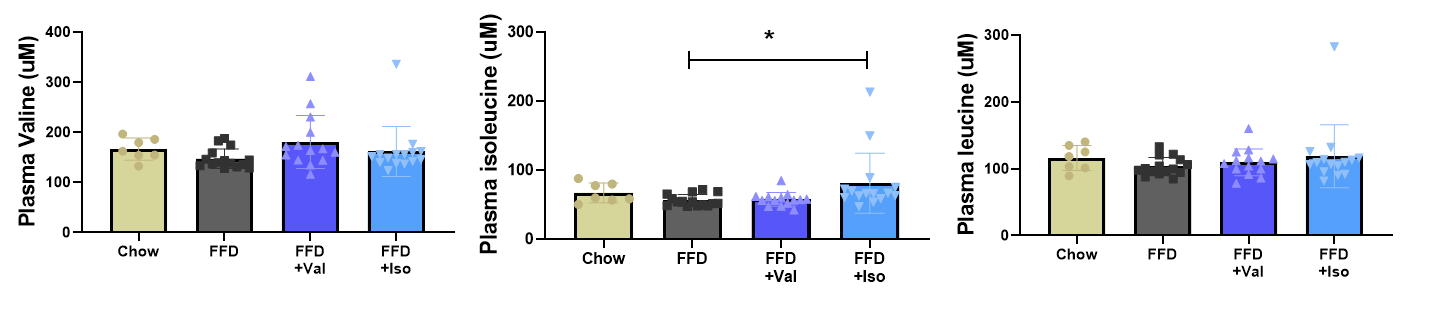
**

**Supplemental figure 1**. Plasma BCAAs concentrations of valine, leucine and isoleucine measured by LC-MS at t=38 weeks in 5h-fasting plasma. An * asterisk indicates significant difference compared to the FFD control group with p<0.05. Data shown are mean ± SD.

**
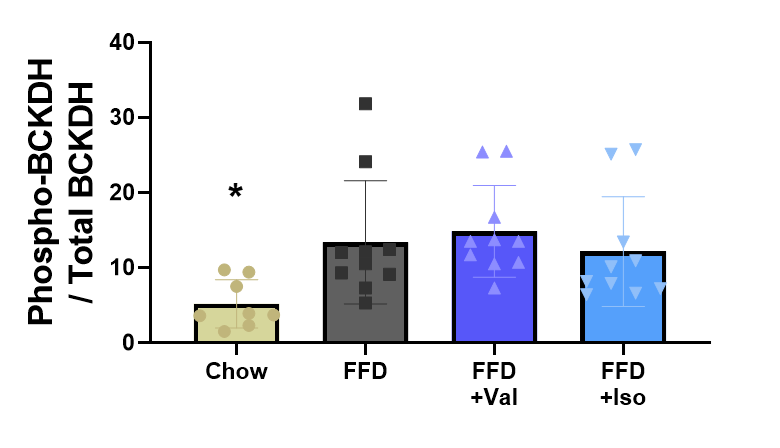
**

**Supplemental figure 2**. Phosphorylated-BCKDH/total-BCKDH protein ratio normalized for tubulin analyzed with Western blots at t=38 weeks. An * asterisk indicates significant difference compared to the FFD control group with p<0.05. Data shown are mean ± SD.

***
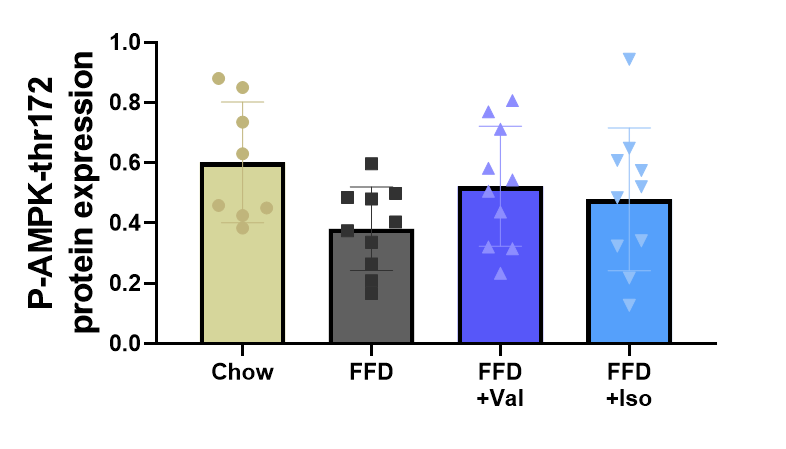
***

**Supplemental figure 3**. Phosphorylated-AMPK normalized for tubulin protein expression analyzed with Western blots at t=38 weeks. An * asterisk indicates significant difference compared to the FFD control group with p<0.05. Data shown are mean ± SD.


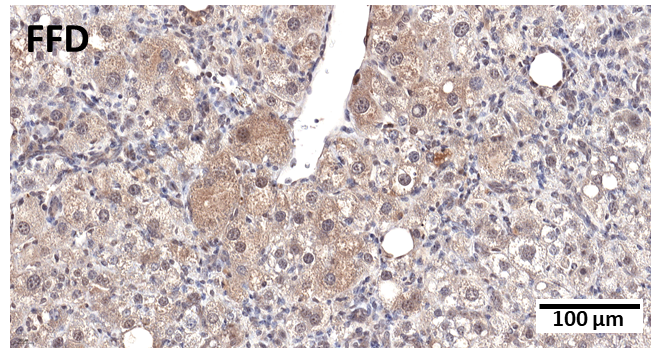


**Supplemental figure 4**. Illustrative photomicrographs of 4-HNE-positive immunoreactivity in enlarged hepatocytes.
